# In vitro antioxidant and anti-diabetic analysis of Andrographis echioides and Andrographis paniculata ethanol extract

**DOI:** 10.6026/97320630018337

**Published:** 2022-04-30

**Authors:** Chevuru Sai Shreya Reddy, Gayatri Devi Ramalingam, J Selvaraj, A Jothi Priya

**Affiliations:** 1 Saveetha Dental College, Saveetha Institute of Medical and Technical Science (SIMATS), Saveetha University, Chennai, India

**Keywords:** Diabetes, antioxidant, Andrographis echioides and Andrographis paniculata, Innovative techniques

## Abstract

It is of interest to analyse and compare the antioxidant and anti-diabetic activity of ethanolic extracts of Andrographis echioides and Andrographis paniculata. Andrographis echioides and Andrographis paniculata were collected from a local farm. In vitro antioxidant activity was assessed by the potential of Piperine, Lupeol, beta sitosterol; DPPH free radical scavenging assay was performed by Liyana Pathirana and Shahidi method. In vitro anti-diabetic activity was assessed by alpha amylase inhibitory activity and alpha glucosidase inhibitory activity. The data were analysed by one-way-ANOVA to check the statistical significance among the groups and considered at the levels of p<0.05. Both the ethanolic extracts of Andrographis echioides and Andrographis paniculata showed significant antioxidant and anti-diabetic potential in a dose-dependent manner (100-500µg) and can be used as potential antidiabetic agents. Similar to antioxidant potential, Andrographis paniculata exhibited an increased anti-diabetic potential compared to Andrographis echioides. Data shows that the ethanolic extracts of Andrographis echioides and Andrographis paniculata possessed antioxidant and anti-diabetic activity and hence our present findings conclude that both plants can be considered for the development of natural drugs for the management of diabetes.

## Background:

The number of people suffering with diabetes in India has increased from 26 million in 1990 to 65 million in 2016 [1]. The species of these plants may represent a source of new hypoglycemic compounds for developing better remedies to treat diabetic patients without serious side effects [2]. The oxygen which is indispensable for life, under uncertain conditions has harmful effects on the human body [3]. Most of the harmful effects of oxygen are due to formation and activation of certain chemical compounds, known as ROS, which have a tendency to donate oxygen to other compounds [4]. Free radicals and antioxidants have become commonly used terms in modern discussion and relatability of disease mechanisms [5]. Andrographis echioides and Andrographis paniculata were given much attention in recent times, because of the therapeutics, pharmaceutical and health protective value as potential plants for treating the Dengue fever [6]. Hence, it has a very high demand and usage in international markets. Andrographolide, Dehydroandrographolide, Neoandrographolide and deoxyandrographolide are the main bioactive compounds present in these plants [7]. Andrographis echioides is located in the dry lands of South Asian countries. Andrographis paniculata is used as a herbal medicine in India, Bangladesh, China and Hong Kong [8,9]. Andrographis paniculata has a broad range of pharmacological effects including anticancer, antidiarrheal, anti hepatitis, anti microbial, anti malarial, and antioxidant activities [10]. Our team has extensive knowledge and research experience that has translated into high quality publications [11- 13, 14-19, 20-30]. Recent demand has increased for Andrographis echioides and Andrographis paniculata which led to the research of these plants in treating various diseases. Therefore, it is of interest to document the antioxidant and antidiabetic activity of Andrographis echioides and Andrographis paniculata ethanol extracts were compared in vitro.

## Materials and methods:

### Chemicals:

All chemicals and reagents used for this research work were purchased from Sigma Chemical Company St. Louis, MO, USA; Invitrogen, USA; Eurofins Genomics India Pvt Ltd, Bangalore, India; New England Biolabs (NEB), USA.

### Collection of plant material:

Andrographis echioides and Andrographis paniculata were collected from Chennai District, Tamil Nadu, India. The species were identified and authenticated at the Department of Centre for Advanced Study in Botany, University of Madras, and Chennai, India. The bark leaves and flower parts of the plant were shade-dried, cut into small pieces and coarsely powdered. The coarse powder was used for extraction with ethanol.

### Preparation of plant extracts:

1kg of dry powders from leaves from both plants was taken in individual aspirator bottles; 3 liters of ethanol was used and the mixture was shaken occasionally for 72 hours. Then the extract was filtered. This procedure was repeated three times and all extracts were decanted and pooled. The extracts were filtered before drying using whatman filter paper no 2 on a Buchner funnel and the solvent was removed by vacuum distillation in a rotary evaporator at 40°C; the extracts were placed in pre-weighed flasks before drying.

### Assessment of in vitro anti-diabetic activity by plant extract Inhibition of albumin denaturation:

The anti-diabetic activity of the plant extract was studied by the inhibition of albumin denaturation technique which was studied according to the methods of Mizushima and Kobayash, 1968 and Sakat et al. (2010) followed with minor modifications. The reaction mixture consisted of test extracts and 1% aqueous solution of bovine albumin fraction, pH of the reaction mixture was adjusted using a small amount of 1N HCl. The plant extract with increase in concentration (100 to 500 µg/ml) were incubated at 37°C for 20 min and then heated to 51°C for 20 min, after cooling the samples the turbidity was measured at 660nm.(UVVisible Spectrophotometer Model 371, Elico India Ltd) The experiment was performed in triplicate. In this study, Aspirin was used as a standard anti-diabetic drug. Calculation: % Inhibition=100-((A1 -A2)/A0)*100). 

### Statistical analysis:

The data were analysed statistically using one way analysis of variance (ONE-WAY ANOVA). Duncan Multiple range test was used to analyze the statistical significance between groups. The levels of significance were considered at the levels of p<0.05.

## Results:

### Alpha amylase inhibitory activity of Andrographis echioides 

In the present study, Andrographis echioides significantly (p<0.05) increased the Alpha amylase inhibitory activity in a dose-dependent manner (100-500µg/ml). However, 400 and 500µg concentrations exhibited the maximum activity in inhibiting the alpha amylase (Figure 1 - see PDF), suggesting that the plant has potential antidiabetic activity.

### Alpha amylase inhibitory activity of Andrographis paniculata:

In the present study, Andrographis paniculata significantly (p<0.05) increased the Alpha amylase inhibitory activity in a dose-dependent manner (100-500µg/ml). However, 400 and 500µg concentrations exhibited the maximum activity in inhibiting the alpha amylase (Figure 2 - see PDF), suggesting that the plant has potential antidiabetic activity.

### Alpha glucosidase inhibitory activity

Alpha glucosidase inhibitory activity of Andrographis echioides

In the present study, Andrographis echioides significantly (p<0.05) increased the Alpha glucosidase inhibitory activity in a dose-dependent manner (100-500µg/ml). However, 400 and 500µg concentrations exhibited the maximum activity in inhibiting the alpha amylase (Figure 3 - see PDF), suggesting that the plant has potential antidiabetic activity.

### Alpha glucosidase inhibitory activity of Andrographis paniculata:

In the present study, Andrographis paniculata significantly (p<0.05) increased the Alpha glucosidase inhibitory activity in a dose-dependent manner (100-500µg/ml). However, 400 and 500µg concentrations exhibited the maximum activity in inhibiting the alpha amylase (Figure 3 - see PDF), suggesting that the plant has potential anti-diabetic activity.

### In vitro antioxidant activity

DPPH radical scavenging activity of Andrographis echioides

In the present study, Andrographis Andrographis echioides significantly (p<0.05) increased the DPPH radical scavenging activity in a dose-dependent manner (100-500µg/ml). However, 400 and 500µg concentrations exhibited the maximum activity in inhibiting the alpha amylase (Figure 5 - see PDF), suggesting that the plant has potential antidiabetic activity.

### DPPH radical scavenging activity of Andrographis paniculata

Data shows that Andrographis paniculata significantly (p<0.05) increased the DPPH radical scavenging activity in a dose-dependent manner (100-500µg/ml). However, 400 and 500µg concentrations exhibited the maximum activity in inhibiting the alpha amylase (Figure 6 - see PDF), suggesting that the plant has potential antidiabetic activity.

## Discussion:

Antioxidant activity of aqueous seed extract of Andrographis echioides and Andrographis paniculata was determined by DPPH free radical scavenging assay. Free radicals / molecules possessing an unpaired electron lead to oxidative stress. The effects of the antioxidants on DPPH free radical scavenging was considered to be due to their hydrogen donating ability. The results obtained in the study show that both the species exhibit significant antioxidant activity as compared with the standard Vitamin C. Andrographis paniculata significantly exhibited a higher antioxidant activity as compared to the Andrographis echioides [31 - check with author]. Further studies may be needed to find out the potential health benefits of the extracts in prevention and scavenging of free radicals. In the study, in vitro α-amylase inhibitory activity and α glucosidase inhibitory activity of extracts of Andrographis echioides and Andrographis paniculata were studied. Results revealed a dose dependent increase in % of inhibitory activity. The extracts showed potent anti diabetic activity in a dose dependent manner, with comparison and increased in a dose dependent manner as compared to the standard. There is a positive relationship between the extracts and ability to inhibit α-glucosidase and α-amylase [32]. Outcome of this study indicates that extracts of Andrographis echioides and Andrographis paniculata could be used as potential antidiabetic agents [33 - check with author]. Similar to antioxidant potential, Andrographis paniculata exhibited an increased anti-diabetic potential compared to Andrographis echioides. In Andrographis echioides, the whole plant is beneficial in controlling the blood glucose level and reducing or preventing diabetes. Andrographis echioides improves the lipid metabolism and prevents diabetic complications from lipid peroxidation and antioxidant systems. This could be useful for prevention or early treatment of diabetic disorders [34]. Ethanol extract of this plant possesses significant antihyperglycemic, antihyperlipidemic, and antioxidant effects in alloxan induced diabetic rats. Andrographis echioides, the whole plant parts - leaf, stem, roots, all are beneficial in controlling blood sugar and can be used in treatment of diabetes. The Andrographis paniculata can be used in treatment in reducing sugar levels, controlling diabetes [35]. Andrographis paniculata is potentially developed as an alternative anti-diabetic agent [36]. Andrographis paniculata give the best anti-diabetic activity to treat obese diabetic conditions [37]. The effects of combination of extracts from Andrographis paniculata [Burm .f.] Nees and Azadirachta indica A is known [38]. Just blood glucose levels were lowered more efficiently than compared to single extract Combination has potential to develop as an anti-diabetic agent. The aqueous and ethanolic extracts of Andrographis paniculata are capable of exhibiting significant blood sugar lowering effects [2]. Andrographis echioides, the whole plant exhibits antioxidant properties. Different extracts of Andrographis echioides can be very effective antioxidants and it could protect biological systems against the oxidative stress including ageing, cancer, diabetes and cardiovascular disorder. Andrographis echioides is a good natural antioxidant source and has higher antioxidant potential [39]. Andrographis paniculata possess antioxidant properties and can be used in treating various disorders. Andrographis paniculata leaves possess anti-inflammatory, antioxidant and analgesic properties [40]. The components get involved in the antioxidant activity of Andrographis paniculata [41]. The antioxidant property can be delayed and prevent oxidation process and forms readily oxidizable substrates and it reduces to low concentration. It is used in lung infection treatments. A limitation of the study is that the sample size cannot be generalised over a population of a particular area. Sample size is very less as we are confined to few samples of the plant.

## Conclusion:

Data shows that Andrographis echioides and Andrographis paniculata possess both antioxidant and anti-diabetic properties in ethanol extracts. The plant species is useful in treatment of various disorders and in prevention of diabetes without any side effects and with the natural properties of the plant.
